# Fabricating Mechanically Robust Binder‐Free Structured Zeolites by 3D Printing Coupled with Zeolite Soldering: A Superior Configuration for CO_2_ Capture

**DOI:** 10.1002/advs.201901317

**Published:** 2019-07-01

**Authors:** Shuang Wang, Pu Bai, Mingzhe Sun, Wei Liu, Dongdong Li, Wenzheng Wu, Wenfu Yan, Jin Shang, Jihong Yu

**Affiliations:** ^1^ State Key Laboratory of Inorganic Synthesis and Preparative Chemistry College of Chemistry Jilin University Changchun 130012 China; ^2^ School of Energy and Environment City University of Hong Kong Tat Chee Ave Kowloon Hong Kong China; ^3^ School of Mechanical and Aerospace Engineering Jilin University Changchun 130025 China; ^4^ Key Laboratory of Automobile Materials of MOE Department of Materials Science and Engineering Jilin University Changchun 130012 China; ^5^ International Center of Future Science Jilin University Changchun 130012 China

**Keywords:** 3D printing, CO_2_ capture, hydrothermal crystallization, monoliths, zeolites

## Abstract

3D‐printing technology is a promising approach for rapidly and precisely manufacturing zeolite adsorbents with desirable configurations. However, the trade‐off among mechanical stability, adsorption capacity, and diffusion kinetics remains an elusive challenge for the practical application of 3D‐printed zeolites. Herein, a facile “3D printing and zeolite soldering” strategy is developed to construct mechanically robust binder‐free zeolite monoliths (ZM‐BF) with hierarchical structures, which can act as a superior configuration for CO_2_ capture. Halloysite nanotubes are employed as printing ink additives, which serve as both reinforcing materials and precursor materials for integrating ZM‐BF by ultrastrong interfacial “zeolite‐bonds” subjected to hydrothermal treatment. ZM‐BF exhibits outstanding mechanical properties with robust compressive strength up to 5.24 MPa, higher than most of the reported structured zeolites with binders. The equilibrium CO_2_ uptake of ZM‐BF reaches up to 5.58 mmol g^−1^ (298 K, 1 bar), which is the highest among all reported 3D‐printed CO_2_ adsorbents. Strikingly, the dynamic adsorption breakthrough tests demonstrate the superiority of ZM‐BF over commercial benchmark zeolites for flue gas purification and natural gas and biogas upgrading. This work introduces a facile strategy for designing and fabricating high‐performance hierarchically structured zeolite adsorbents and even catalysts for practical applications.

Selective separation and capture of CO_2_ by a cost‐effective and energy‐efficient technology has always been a research hotspot, because of the multiple roles of CO_2_ as a greenhouse gas, renewable carbon source, and even as raw material to produce liquid fuels.^1^, ^2^ In addition, CO_2_ is also an impurity in enclosed environments, natural gas, biogas, landfill gas, syngas, and many other gas mixtures.^3^ Crystalline aluminosilicate zeolites with molecular dimensional micropores, large surface area, excellent thermal and chemical stability, and internal electrical field gradients are popular adsorbents for CO_2_ capture and other gas separation.^4^, ^5^ Particularly, NaX zeolite is extensively used in CO_2_ capture due to its low SiO_2_/Al_2_O_3_ ratio, cheap price, and high adsorption capacity.^6^, ^7^

Traditionally, the primary nano or micrometer‐sized zeolite crystals are agglomerated into macroscopic (mm) secondary structures (e.g., granules, pellets, and extrudates) to cater for industrial applications.^8^ To overcome the intrinsic limitations of conventional‐shaped adsorbents (or catalysts), such as slow mass transfer or heat transfer, excessive pressure drop, and poor attrition resistant, it is more desirable to fabricate zeolite powders into hierarchically structured zeolites with an optimized configuration.^9^ So far, two categories of conventional strategies have been developed for designing and manufacturing structured zeolites. One is depositing active zeolite powders onto the surface of preprocessed macroporous supports, such as ceramic foams,^10^ porous titanium alloy,^11^ and cordierite.^12^ Unfortunately, employing some of these porous supports obviously reduces the practical volume efficiency, especially in the case of a very low zeolite loading.^13^, ^14^ Self‐supporting is the other strategy through freeze casting,^15^ sacrificial templating,^16^ quasi‐solid gel crystallization,^17^ pulsed current processing,^18^ which is an effective approach to enhance the volume efficiency of the fixed bed reactor. However, subjected to the inflexible configuration, complicated manufacturing process, and poor mechanical integrity, this strategy still faces many obstacles for their large‐scale implementation.^13^, ^18^, ^19^

Recently, the unique capabilities of computer‐aided additive manufacturing, also known as 3D printing, for accurate fabrication of geometries with customization, flexibility, and complexity, drive a revolution in the fields of biomedical engineering, energy, catalysis, and environment.^20^, ^21^, ^22^, ^23^ Various porous materials including porous ceramics,^24^ porous polymers,^25^ metal–organic frameworks,^26^ and covalent organic frameworks^27^ with complex self‐supporting architectures have been successfully fabricated by 3D printing, particularly, 3D printing has proven to be an attractive strategy to tailor monolithic zeolite adsorbents and catalysts with hierarchical structures that are favorable for diffusions.^28^, ^29^, ^30^, ^31^ However, due to the difficulty of integrating individual zeolite crystals with robust interfacial binding, the practical use of 3D‐printed structured zeolites is severely restricted by their insufficient mechanical strength.^32^, ^33^ For instance, in fixed bed reactors, structured zeolites must withstand frequent and long‐time pressure changes; although the incorporation of some printing ink additives (e.g., organic or inorganic binders) can improve the mechanical stability to some extent, it inevitably results in diffusion limitation, partial pore blocking, and dilution of the active zeolites.^13^, ^34^ The trade‐off among mechanical strength, mass loading of active zeolites, and diffusion kinetics remain a challenging hurdle for the practical application of 3D‐printed structured zeolites.^33^, ^35^, ^36^ Thus, it is highly desirable to develop a facile strategy to fabricate 3D‐printed binder‐free hierarchically structured zeolites with merit of fusing mechanical robustness, fast mass diffusion, and high zeolite loading for the practical pressure/temperature swing adsorption.

The characteristics of printing ink additives have a significant influence on the rheological properties and extrusion abilities of printing inks and the physical and chemical properties of the final configuration.^32^, ^37^ Halloysite nanotubes (HNTs), with the molecular formula of Al_2_Si_2_O_5_(OH)_4_·*n*H_2_O, are natural 1D materials with a unique tubular microstructure.^38^ Owing to the unique nano‐tubular structure, natural availability, and low cost, HNTs have been widely applied in ceramics, drug sustained release system, catalysis, and adsorption.^39^, ^40^, ^41^ Furthermore, HNTs are also excellent mechanical reinforcing materials alternative to the expensive carbon nanotubes for numerous polymers, because of their high aspect ratio, unique surface properties, and high mechanical strength (the Young's modulus is as high as 140 GPa).^42^, ^43^ The unique nature of HNTs motivates us to hypothesize that the incorporation of HNTs as a printing ink additive in the fabrication of structured zeolites may lead to a robust 3D network. More encouragingly, thanks to the unique elemental composition (SiO_2_/Al_2_O_3_ = 2), HNTs could be used as an ideal precursor material to synthesize zeolites with low SiO_2_/Al_2_O_3_ ratios, such as zeolite A (SiO_2_/Al_2_O_3_ = 2) and X (SiO_2_/Al_2_O_3_ = 2–3), by post‐hydrothermal treatment.^44^, ^45^ Importantly, the electrostatic interactions between negatively charged aluminosilicate framework and charge‐balancing cation in such low‐silica zeolites provide abundant adsorption sites for the high‐boiling point and quadrupolar CO_2_ molecule.^46^ We expect that the combination of 3D printing with appropriate post‐processing will promise the properties of structured zeolites and drive the innovation of 3D printing technology in zeolites manufacturing process.

In this proof‐of‐concept study, a facile and versatile “3D printing and zeolite soldering” strategy has been developed to manufacture binder‐free NaX zeolite monoliths (ZM‐BF) with robust mechanical integrity, hierarchical structure, and outstanding CO_2_ adsorption capacity. In this strategy, for the first time, we introduced HNTs as a printing ink additive for reinforcing the mechanical strength of zeolite monoliths. Upon subsequent hydrothermal crystallization, interfacial “HNTs‐bridges” were transformed into ultrastrong “zeolite‐bonds,” which promised high mechanical strength and fast diffusion kinetics, as well as excellent CO_2_ uptake and selectivity of the fabricated ZM‐BF. Especially, at low partial pressure, ZM‐BF displayed an ultrastrong CO_2_ affinity compared with the parental NaX zeolite powders, which was more favorable for trace CO_2_ capture. Strikingly, the column breakthrough mixed gas experiments proved that ZM‐BF had excellent dynamic separation performance compared with conventional‐shaped benchmark zeolites. The successful fusion of emerging 3D printing technology with traditional hydrothermal crystallization provides a facile strategy for fabricating high‐performance binder‐free structured zeolites in gas separation, as well as in catalysis, sensoring, and other advanced applications.

Commercial NaX zeolite powders (SiO_2_/Al_2_O_3_ = 2.37) with narrow crystal size distributions (2–3 µm) and a well‐defined morphology (Figure S1, Supporting Information), were adopted to manufacture 3D‐printed zeolite monoliths. HNTs (SiO_2_/Al_2_O_3_ = 2.02) introduced as a printing ink additive exhibit a typical nano‐tubular structure with a diameter of 30–50 nm and a length of 0.2–1 µm (aspect ratio of 4–40) (Figure S2, Supporting Information). As illustrated in the schematic diagram (**Figure** **1**), the procedure for fabricating 3D‐printed mechanically robust binder‐free zeolite monoliths mainly involves in four steps: I) preparing homogenous zeolites containing inks, II) printing pristine zeolite monoliths (denoted as ZM‐P), III) high‐temperature calcination treatment to obtain zeolite monoliths with binder of HNTs (denoted as ZM‐WB), and IV) hydrothermal crystallization to transform ZM‐WB into binder‐free zeolite monoliths (denoted as ZM‐BF). The overall size of the monolith as well as rod diameter and rod spacing can be adjusted by the nozzle size and program used during the printing. By using this facile strategy, 3D‐printed binder‐free zeolite monoliths with tailorable geometries (**Figure** **2**a and Figure S3 and Table S1, Supporting Information) and robust mechanical stability (Figure 2b) were successfully fabricated. Notably, the channels per square inch (cpsi) of S4 in Figure 2a is as high as 645 (Figure S3 and Table S1, Supporting Information).

**Figure 1:**
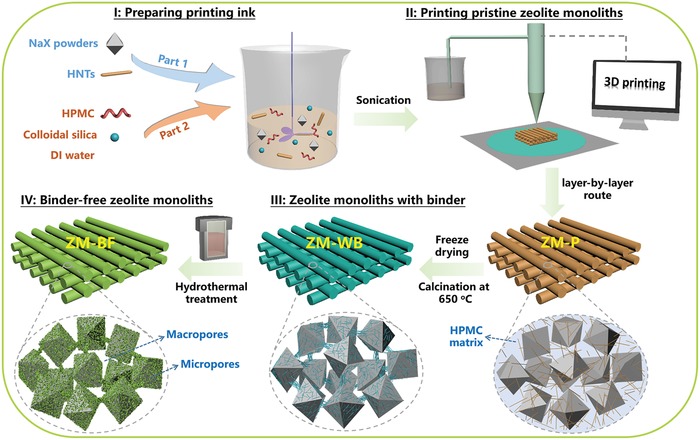
Schematic diagram of fabrication procedure of 3D‐printed mechanically robust binder‐free zeolite monoliths: I) a homogenous printing ink with optimal viscosity was obtained by mixing “part 1” and “part 2” including NaX powders, HNTs, HPMC, colloidal silica, and deionized water. II) The zeolites containing inks extruded through a nozzle in a 3D printing system followed the well‐designed movement controlled by a computer, resulting in ZM‐P. III) High‐temperature calcination treatment to obtain ZM‐WB. IV) Hydrothermal crystallization to produce ZM‐BF.

**Figure 2:**
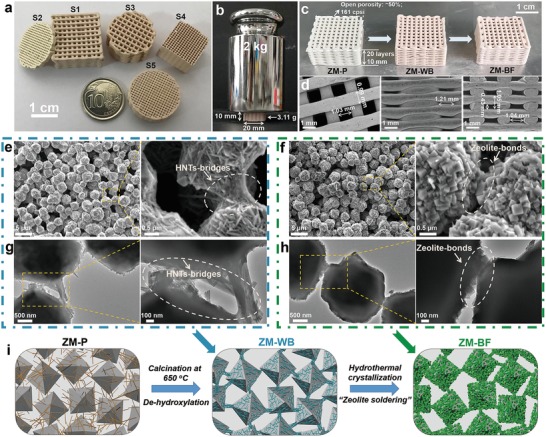
a,b) Digital photographs of ZM‐BF with tailorable geometries and robust mechanical stability. c) Digital photographs of ZM‐P, ZM‐WB, and ZM‐BF. d) Low‐magnification SEM images of a representative ZM‐BF: top view, side view, and cross‐sectional view. e–h) High‐magnification SEM images and TEM images of the cross‐section of ZM‐WB (e,g) and ZM‐BF (f,h). i) The schematic of the procedure of zeolite soldering.

Specifically, homogenous zeolites containing inks with suitable rheological properties were first prepared from mixtures with a typical formulation (except water) of *x* wt% NaX zeolites, *y* wt% HNTs (*x* + *y* = 97, *y* = 0, 7, 14, 21), 1 wt% silica (added in the form of colloidal silica), and 2 wt% hydroxypropyl methylcellulose (HPMC, plasticizing agent). The HNTs content has a significant influence on the rheological properties of printing inks (Figure S4, Supporting Information). The viscosity of the printing inks increased with the increase of the HNTs content. However, the printing ink was too viscous to achieve extrusion by the pneumatic‐injection 3D printing system when the HNTs content reached to 21 wt%. Although the low HNTs content (0 and 7 wt%) is beneficial for extrusion, the mechanical stability of the obtained 3D‐printed zeolite monolith was poor (Figure S5, Supporting Information). The 3D‐printed zeolite monolith fabricated without HNTs additives has the compressive strength of only 0.17 MPa. Therefore, the printing ink with 14 wt% HNTs was used (the formulation of printing inks was 83 wt% NaX zeolites, 14 wt% HNTs, 1 wt% silica, and 2 wt% HPMC) in the following studies. The addition of appropriate amount of colloidal silica as extra silicon source was to inhibit the transformation of HNTs into zeolite A.^44^ The mixture was stirred thoroughly for 2 h, followed by sonication to ensure homogeneity and avoid air bubbles. By using the above printing inks, a nozzle settled at a 3D printer was employed to print predesigned geometry via a layer‐by‐layer route. The as‐prepared ZM‐P was then freeze‐dried to prevent partial shrink and collapse, followed by calcination at 650 °C to enhance the mechanical stability and form abundant macropores by removing the HPMC matrix. During calcination, the dispersed HNTs experienced a de‐hydroxylation process, which could lead to enhanced interaction between HNTs and NaX zeolite particles. Afterward, the resultant ZM‐WB was transferred into 3 m sodium hydroxide solution and then crystallized at 95 °C for 8 h in an oven to obtain ZM‐BF. Notably, the structured zeolites retained the printed shape after high‐temperature calcination treatment and further hydrothermal crystallization (Figure 2c), demonstrating their outstanding overall structural integrity. Scanning electron microscopy (SEM) images of the representative ZM‐BF honeycomb structure show that the 3D‐printed monolith preserves internal structural integrity and the interconnected rods have a smooth exterior surface and a uniform dimension without deformation (Figure 2d).

Figure 2e,f shows the magnified SEM images of ZM‐WB and ZM‐BF. The high‐magnification SEM images of the cross‐section of ZM‐WB (Figure 2e) show that the HNTs are adhered to the external surface of primary NaX zeolite crystals. The formation of “HNTs‐bridges” on the NaX zeolites interface boundary results in a well‐developed interconnected 3D network with open macroporosity. Strikingly, continuous layers comprised of particles with size of about 100–300 nm appeared and tightly wedged around the surface of NaX zeolite crystals after hydrothermal crystallization (Figure 2f), indicating the phase transformation of HNTs into zeolites. To gain further insight into the microscopic changes over the surface of NaX zeolite crystals, ZM‐WB and ZM‐BF were investigated via transmission electron microscopy (TEM; Figure 2g,h). Although the zeolite monoliths were ground and sonicated, the NaX zeolite particles in ZM‐WB remained closely knitted together by HNTs, forming strong “HNTs‐bridges” (Figure 2g). The “HNTs‐bridges” further transformed into robust “zeolite‐bonds” after hydrothermal crystallization (Figure 2h). Such “zeolite‐bonds” in the interconnected network is expected to play a key role in reinforcing the mechanical stability of ZM‐BF. The newly formed robust “zeolite‐bonds” on the interface boundary of the primary NaX zeolites resemble the soldering joints during metal manufacture, so we refer to this process as “zeolite soldering.” Figure 2i illustrates the proposed “zeolite soldering” process for manufacturing robust and binder‐free 3D‐printed zeolite monoliths.

**Figure** **3**a shows the powder X‐ray diffraction (PXRD) patterns of ZM‐WB and ZM‐BF, along with the parental NaX powders. All the characteristic diffraction peaks of NaX zeolite are retained in both ZM‐WB and ZM‐BF. The relative crystallinity of ZM‐WB is 69.3% of the parental NaX powders. The decreased peak intensity of ZM‐WB results from the incorporation of HNTs and colloidal silica, and the high temperature calcination.^33^ After hydrothermal crystallization, the relative crystallinity of ZM‐BF increases to 92.5% with respect to the parental NaX powders. The peak intensities for ZM‐BF obviously increase owing to the transformation of HNTs and colloidal silica into zeolite NaX. It is noticeable that weak diffraction peaks of zeolite NaA (SiO_2_/Al_2_O_3_ = 2) appear in ZM‐BF. However, the fraction of zeolite NaA in ZM‐BF would increase substantially if no colloidal silica was added in the printing inks (Figure S6, Supporting Information). Notably, colloidal silica employed as extra silicon source could significantly inhibit the transformation of HNTs to NaA, which is unfavorable for CO_2_ capture.^47^, ^48^

**Figure 3:**
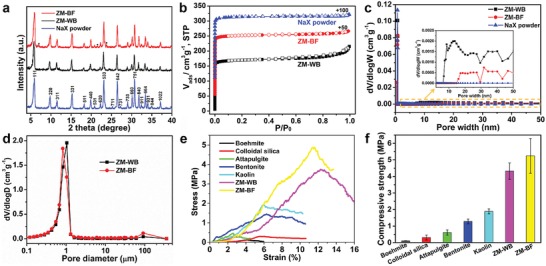
Physical and microstructural characterizations of 3D‐printed zeolite monoliths. a) PXRD patterns of NaX zeolite powder, ZM‐WB, and ZM‐BF. Note: peaks marked with black dot are the characteristic diffraction peaks of zeolite NaA. b) N_2_ adsorption–desorption isotherms of the NaX powder, ZM‐WB, and ZM‐BF. c) The PSD curves of the NaX powder, ZM‐WB, and ZM‐BF derived from DFT method. d) PSD curves of ZM‐WB and ZM‐BF determined by mercury intrusion porosimetry. e,f) Representative stress–strain curves (e) and compressive strength (f) of 3D‐printed zeolite monoliths fabricated with various printing ink additives.

The textural properties of ZM‐WB, ZM‐BF, and NaX zeolite powders were assessed by N_2_ adsorption–desorption isotherms and mercury intrusion porosimetry. The N_2_ adsorption–desorption isotherms of all materials show sharp uptakes at *P*/*P*_0_ < 0.1, which is characteristic of microporous zeolites (Figure 3b). The appearance of a hysteresis loop at *P*/*P*_0_ = 0.75–1.0 in ZM‐WB is mainly attributed to the intrinsic mesoporous structure of HNTs (Figure S7, Supporting Information) and the macropores formed after the removal of HPMC matrix upon calcination. Significantly, the N_2_ isotherm of ZM‐BF displays a higher N_2_ uptake than ZM‐WB due to the transformation of HNTs into zeolite crystals. The density functional theory (DFT) method was employed for estimating the pore size distributions (PSDs). The peaks at 0.6–0.8 nm correspond to micropores in NaX for all of the samples (Figure 3c). The wide PSDs in the range 2–50 nm indicate that both ZM‐WB and ZM‐BF possess mesopores structures (the inset of Figure 3c). The detailed surface area and pore volume data are summarized in Table S2 in the Supporting Information. ZM‐BF displays significantly increased micropore surface (*S*_micro_ = 571 m^2^ g^−1^) and micropore volume (*V*_micro_ = 0.295 cm^3^ g^−1^) as compared with ZM‐WB (*S*_micro_ = 470 m^2^ g^−1^ and *V*_micro_ = 0.242 cm^3^ g^−1^). The external surface area and mesopore volume of ZM‐BF are 40 m^2^ g^−1^ and 0.040 cm^3^ g^−1^, respectively, which are higher than the parental NaX powders (*S*_ext_ = 32 m^2^ g^−1^ and *V*_mes_ = 0.017 cm^3^ g^−1^), as a result of the mesoporous character of ZM‐BF. Complementary assessment of macropores size distributions was performed by mercury intrusion porosimetry for both ZM‐WB and ZM‐BF (Figure 3d). In accordance with the SEM observations, the micrometer‐sized macroholes (about 1 µm) are caused by the agglomeration of interconnected zeolite NaX particles (2–3 µm). The minor peaks situated around 91 µm correspond to the macroholes in the monolith wall (Figure S8, Supporting Information), which may be generated by trace air bubbles in the printing inks. Integrated with the intrinsic honeycomb‐based channels, the 3D‐printed zeolite monoliths (both ZM‐WB and ZM‐BF) exhibit a highly interconnected and hierarchical porous structure, which is expected beneficial for CO_2_ diffusion.^13^, ^49^

The mechanical property of 3D‐printed adsorbents is one of the key criteria for their practical application. To evaluate the mechanical stability of printed zeolite monoliths, five types of commercial binders (boehmite, colloidal silica, attapulgite, bentonite, and kaolin) were used as printing ink additives for comparison (Figure S9, Supporting Information). Then, compressive strength tests were performed for different zeolite monoliths and the representative examples of their stress–strain curves are shown in Figure 3e. The Young's modulus of ZM‐BF (42.8 MPa) is much higher than that of ZM‐WB (29.4 MPa), ZM@kaolin (32.5 MPa), ZM@bentonite (22.4 MPa), ZM@attapulgite (17.5 MPa), ZM@colloidal silica (5.49 MPa), and ZM@boehmite (2.89 MPa) (Table S3, Supporting Information). The strain of ZM@boehmite, ZM@colloidal silica, ZM@attapulgite, ZM@bentonite, ZM@kaolin, ZM‐WB, and ZM‐BF at the time of fracture is 4.2%, 5.7%, 2.6%, 6.3%, 5.9%, 12.3%, and 11.4%, respectively (Table S3, Supporting Information). The substantially elevated fracture strains indicate both ZM‐WB and ZM‐BF perform relatively better ductility. Moreover, the compressive strength of ZM‐WB (4.32 MPa) is much higher than that of other zeolite monoliths with commercial binders such as boehmite (0.11 MPa), colloidal silica (0.31 MPa), attapulgite (0.62 MPa), bentonite (1.28 MPa), and kaolin (1.89 MPa) (Figure 3f). Note that kaolin and HNTs have the same element composition, but the compressive strength of ZM@kaolin was obviously lower than ZM‐WB. The above results demonstrate that the unique structure of HNTs is crucial for the reinforcement of printed zeolite monoliths. The reinforcement mechanism of ZM‐WB is mostly attributed to the introduced HNTs with high aspect ratio, nano‐tubular structure, and high strength,^43^ and the formed robust interfacial “HNTs‐bridges” due to the de‐hydroxylation process on the interface boundary between HNTs and the primary NaX zeolite crystals (Figure 2e,f).^40^ Remarkably, after hydrothermal crystallization, the compressive strength of resulting ZM‐BF can reach up to 5.24 MPa. The hydrothermal treatment facilitates the transformation of robust “HNTs‐bridges” in ZM‐WB into much stronger “zeolite‐bonds” in the interconnected 3D network of ZM‐BF (Figure 2g,h), thus further enhancing the mechanical integrity of ZM‐BF.

Table S4 in the Supporting Information summarizes the mechanical performance of reported structured zeolites manufactured by 3D printing (0.05–1.54 MPa)^32^, ^33^ or other shaping strategies, such as direct extrusion (2.58 MPa),^50^ sacrificial templating (0.085 MPa),^16^ pulsed current processing (1.6–2.2 MPa),^34^ freeze casting (0.045–1.38 MPa),^15^, ^51^, ^52^ and slip casting (0.21–0.75 MPa).^53^ It is clear that ZM‐BF exhibits a much higher mechanical strength than most of the reported structured zeolites, which is only lower than that of 3D‐printed polymer‐zeolite composite monoliths.^35^ However, the 3D‐printed polymer‐zeolite composite monoliths suffered from severe reduction of the specific surface area (59 m^2^ g^−1^) and CO_2_ adsorption capacity (1.83 mmol g^−1^) due to the heavy use of Torlon polymer and the possible zeolite pore blockage.

Single component equilibrium adsorption isotherms for CO_2_, N_2_, and CH_4_ were measured at 298 K up to 1 bar. As presented in **Figure** **4**a, the CO_2_ uptake of ZM‐WB is 4.49 mmol g^−1^ under 1 bar, which is strongly affected by the presence of HNTs additives, resulting in around 21% reduction with respect to the parental NaX zeolite powders (5.68 mmol g^−1^ under the same condition). As expected, the adsorption capacity of CO_2_ for ZM‐BF increases up to 5.58 mmol g^−1^ at 298 K under 1 bar (6.55 mmol g^−1^ at 273 K, Figure S10, Supporting Information), which is significantly higher than that of ZM‐WB and comparable to that of the parental NaX zeolite powders. To evaluate the CO_2_ uptake performance of ZM‐BF, a series of top‐commercial CO_2_ adsorbents including NaX extrudates (*d* = 1.5 mm, denoted as C‐NaX‐1), NaX pellets (*d* = 1.6–2.5 mm, denoted as C‐NaX‐2), NaX extrudates (*d* = 3 mm, denoted as C‐NaX‐3), and granular activated carbon (*d* = 1–2 mm, denoted as GAC) were also selected for comparison. The CO_2_ uptake of ZM‐BF is obviously superior to these top‐commercial adsorbents. Table S5 in the Supporting Information summarizes the CO_2_ uptake of various structured adsorbents, such as zeolites, porous carbons, aminosilica monoliths, and MOFs. Remarkably, ZM‐BF exhibits a much higher adsorption capacity than most of the reported structured CO_2_ adsorbents and is the highest among all reported 3D‐printed structured CO_2_ adsorbents.

**Figure 4:**
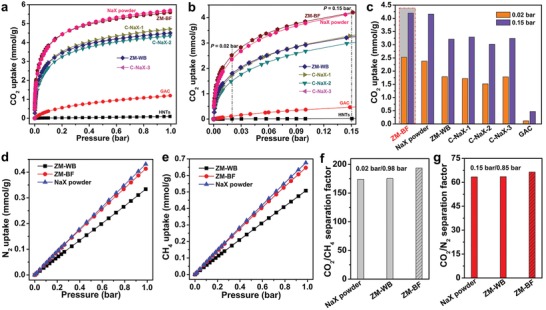
a,b) Comparison of the CO_2_ adsorption isotherms of ZM‐BF, ZM‐WB, NaX powder, C‐NaX‐1, C‐NaX‐2, C‐NaX‐3, and GAC in the *P*_CO__2_ region between 0 and 1 bar (a) and between 0 and 0.15 bar at 298 K (b). c) The CO_2_ uptake of ZM‐BF, NaX powder, ZM‐WB, and commercial adsorbents under 0.02 and 0.15 bar, respectively at 298 K. d) N_2_ and e) CH_4_ adsorption isotherms for NaX powders, ZM‐WB, and ZM‐BF obtained at 298 K. f) CO_2_/CH_4_ separation factors at 0.02/0.98 bar and g) CO_2_/N_2_ separation factors at 0.15/0.85 bar for NaX powder, ZM‐BF, and ZM‐WB.

More encouragingly, ZM‐BF indicates excellent CO_2_ capture capacity at low partial pressures (0 ≤ *P*_CO__2_ ≤ 0.15 bar, Figure 4b). At 0.02 and 0.15 bar, which are relevant to the CO_2_ capture abilities of adsorbents from natural gas (CO_2_/CH_4_:2/98, v/v) and flue gas (CO_2_/N_2_:15/85, v/v), respectively, ZM‐BF exhibits notably enhanced CO_2_ uptake (2.53 mmol g^−1^ at 0.02 bar and 4.20 mmol g^−1^ at 0.15 bar) versus ZM‐WB (1.79 mmol g^−1^ at 0.02 bar and 3.21 mmol g^−1^ at 0.15 bar) (Table S6, Supporting Information). In comparison to other top‐commercial adsorbents, ZM‐BF also exhibits higher CO_2_ uptake than all of the investigated commercial materials (Figure 4c), even higher than the parental NaX powders (2.38 mmol g^−1^ at 0.02 bar and 4.16 mmol g^−1^ at 0.15 bar). These results confirm a very high CO_2_ capture ability of ZM‐BF at low partial pressures, possibly because the recrystallized zeolites with lower SiO_2_/Al_2_O_3_ ratio (SiO_2_/Al_2_O_3_ = 2.26) than the parental NaX powders (SiO_2_/Al_2_O_3_ = 2.37) possess stronger electrostatic field and more adsorption sites (Table S7, Supporting Information).^54^, ^55^ Considering the fact that the CO_2_ concentration in common gas mixtures is relatively low, the higher CO_2_ uptake on ZM‐BF under low partial pressure is more meaningful for CO_2_ capture.

The adsorption capacities of N_2_ and CH_4_ on ZM‐BF also increase compared with ZM‐WB, and are comparable to the parental NaX powders, but they are still much lower than that of CO_2_ uptake (Figure 4d,e). To evaluate the selectivity of ZM‐BF, we calculated the separation factors α of CO_2_/CH_4_ (at 0.02/0.98 bar) and CO_2_/N_2_ (at 0.15/0.85 bar) determined from single‐component isotherms and compared with that of ZM‐WB and the parental NaX powders (Figure 4f,g). Given the observed ultrastrong CO_2_ affinity under low partial pressure, ZM‐BF were found to exhibit an elevated selectivity for binary CO_2_/CH_4_ (2/98, v/v) mixtures (α: 194) and CO_2_/N_2_ (15/85, v/v) mixtures (α: 66.5) compared with ZM‐WB and even the parental NaX powders (Table S6, Supporting Information). These results clearly suggest the superior selectivity of ZM‐BF for the separation of natural gas and flue gas. Such superior selectivity, together with high uptake capacity, makes ZM‐BF a promising candidate for CO_2_ capture from natural gas and flue gas.

These single component and static equilibrium adsorption results prompted us to further evaluate the dynamic separation performance of ZM‐BF in a more realistic separation process. **Figure** **5**a shows the schematic of a fixed bed adsorption column packed with conventional‐shaped zeolites (pellets and extrudates) and 3D‐printed zeolite monoliths. The column breakthrough mixed gas experiments were conducted using binary CO_2_/CH_4_ (2/98, v/v), CO_2_/N_2_ (15/85, v/v), and CO_2_/CH_4_ (50/50, v/v) gas mixtures at 296 K and atmospheric pressure (Schematic S1, Supporting Information), mimicking the industrial process conditions of flue gas, natural gas, and biogas, respectively. To match the size of adsorption column, zeolite monoliths were readily printed into cylinders with a diameter and height of 1 cm, while the nozzle diameter during printing process was kept at 0.51 mm and the off‐set in the *z*‐direction was set to 0.4 mm. Three types of commercial conventional‐shaped NaX zeolites mentioned above were also tested for comparison.

**Figure 5:**
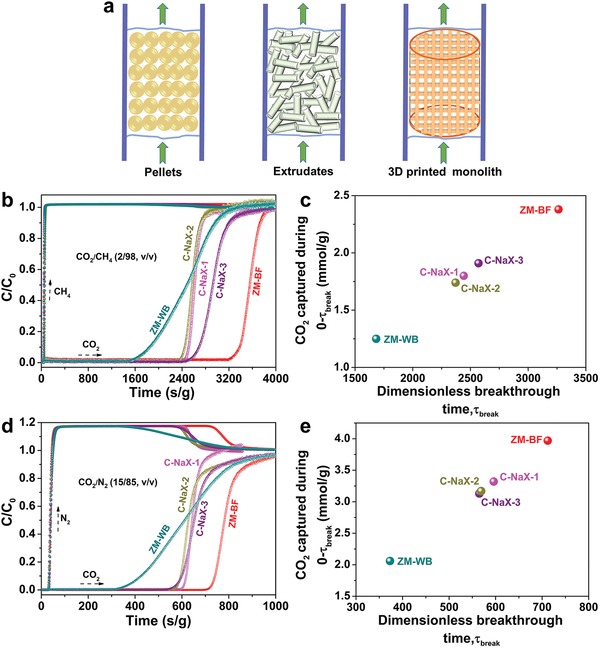
a) Schematic of the packed bed adsorbers with conventional‐shaped zeolites (pellets and extrudates) and 3D‐printed structured zeolites. b,d) The column breakthrough curves for CO_2_/CH_4_ (2/98, v/v) (b) and CO_2_/N_2_ (15/85, v/v) (d) carried out on ZM‐WB and ZM‐BF, and other top‐commercial CO_2_ adsorbents. c,e) Plots of the amount of CO_2_ captured from CO_2_/CH_4_ (c) and CO_2_/N_2_ (e) gas mixtures as a function of τ_break_ in the column breakthrough for ZM‐BF, ZM‐WB, and other commercial benchmark NaX zeolites, where τ_break_ is the breakthrough time at *C*/*C*_0_ = 5%.

As shown in Figure 5b, highly efficient separation for CO_2_/CH_4_ (2/98, v/v) mixture was achieved by ZM‐BF: CH_4_ immediately eluted through the bed in a high‐purity grade, whereas CO_2_ was retained in the packed bed over 3263 s g^−1^ (the CO_2_ concentration in the outlet below 0.05 C_0_). This CO_2_ breakthrough time (τ_break_) on ZM‐BF far exceeds τ_break_ on ZM‐WB (1683 s g^−1^) and conventional‐shaped NaX zeolites (2371–2570 s g^−1^), demonstrating the superior CO_2_ capacity of ZM‐BF. A longer CO_2_ τ_break_ is desirable because of the both reduced regeneration frequency and energy consumption. Figure 5c displays the dynamic adsorption capacity of CO_2_ up to τ_break_ during CO_2_/CH_4_ separation using different adsorbents. During the time 0–τ_break_, the CO_2_ amount captured by ZM‐BF reaches up to 2.38 mmol g^−1^, which is much higher than ZM‐WB (1.25 mmol g^−1^) and commercial benchmark NaX zeolites (1.74–1.91 mmol g^−1^). We further assessed the performance of ZM‐BF for CO_2_ separation from CO_2_/N_2_ (15/85, v/v) mixture (Figure 5d). From the breakthrough curves, τ_break_ of CO_2_ on ZM‐BF was up to 712 s g^−1^, which was significantly longer than that of ZM‐WB (373 s g^−1^) and conventional‐shaped NaX zeolites (565–596 s g^−1^). The dynamic CO_2_ uptake on ZM‐BF (3.97 mmol g^−1^) was also far beyond the value of ZM‐WB (2.06 mmol g^−1^), and conventional‐shaped NaX zeolites (3.13–3.32 mmol g^−1^) (Figure 5e). For equimolar CO_2_/CH_4_ (50/50, v/v) gas mixture, ZM‐BF also exhibits excellent separation performance (Figure S11, Supporting Information). The roll up areas in the breakthrough curves of CH_4_ and N_2_ demonstrated that the adsorbed CH_4_ or N_2_ was replaced by CO_2_, indicating the more affinity of zeolites adsorbents toward CO_2_.^36^ Though the equilibrium CO_2_ uptake of ZM‐WB is comparable to commercial conventional‐shaped NaX zeolites, its breakthrough performance was unsatisfactory. We speculate that the limited CO_2_ diffusion was possibly due to the continuous HNTs coating with poor CO_2_ uptake on the external surface of NaX crystals in ZM‐WB. Significantly, ZM‐BF exhibits much shorter mass‐transfer zone for CO_2_ than ZM‐WB, confirming the faster CO_2_ diffusion kinetics enabled by ZM‐BF. These breakthrough data clearly demonstrate the superior performance of our 3D‐printed binder‐free structured zeolites in selectively separating CO_2_ from flue gas, natural gas, and biogas.

Furthermore, the cycle and regeneration capabilities of ZM‐BF were also successively tested by breakthrough cycle experiments with above all three binary gas mixtures (Figures S12–14, Supporting Information). The dynamic CO_2_ uptake changed within a certain range of error during five continuous cycles, confirming the excellent recyclability of ZM‐BF. The cyclic measurements revealed that ZM‐BF retained the high CO_2_ uptake and selectivity over repeated adsorption–regeneration tests.

In summary, we have developed a facile and versatile “3D printing and zeolite soldering” strategy for fabricating hierarchically structured binder‐free zeolite monoliths, which possess outstanding mechanical properties and high CO_2_ uptake and selectivity. The introduction of HNTs with high aspect ratio, nano‐tubular structure, and high strength as the printing ink additives is beneficial for integrating individual zeolite crystals with robust interfacial “HNTs‐bridges.” Moreover, the successful transformation of HNTs into zeolites contributes to forming the robust interconnected network via “zeolite bonds,” which further enhance the mechanical stability of ZM‐BF. The dynamic breakthrough tests demonstrate the superiority of ZM‐BF over commercial benchmark NaX zeolites for selectively capturing CO_2_ from flue gas, nature gas, and biogas. To the best of our knowledge, this 3D‐printed binder‐free zeolite monolith is the first case of structured zeolites that fully overcomes the trade‐off among mechanical strength, diffusion kinetics, and adsorption capacity. We believe that the “3D printing and zeolite soldering” strategy of current study may afford a versatile pathway for designing and fabricating other binder‐free hierarchically structured zeolites, which may open more advanced applications of 3D‐printed zeolites not only in adsorption but also in other areas such as catalysis and sensoring.

## Conflict of Interest

The authors declare no conflict of interest.

## Supporting information

Supplementary

## References

[1] F. Li , D. R. MacFarlane , J. Zhang , Nanoscale 2018, 10, 6235. 29569672 10.1039/C7NR09620H

[2] M. Bui , C. S. Adjiman , A. Bardow , E. J. Anthony , A. Boston , S. Brown , P. S. Fennell , S. Fuss , A. Galindo , L. A. Hackett , J. P. Hallett , H. J. Herzog , G. Jackson , J. Kemper , S. Krevor , G. C. Maitland , M. Matuszewski , I. S. Metcalfe , C. Petit , G. Puxty , J. Reimer , D. M. Reiner , E. S. Rubin , S. A. Scott , N. Shah , B. Smit , J. P. M. Trusler , P. Webley , J. Wilcox , N. Mac Dowell , Energy Environ. Sci. 2018, 11, 1062.

[3] P. Nugent , Y. Belmabkhout , S. D. Burd , A. J. Cairns , R. Luebke , K. Forrest , T. Pham , S. Ma , B. Space , L. Wojtas , M. Eddaoudi , M. J. Zaworotko , Nature 2013, 495, 80. 23446349 10.1038/nature11893

[4] Y. R. Wang , R. T. Yang , ACS Sustainable Chem. Eng. 2019, 7, 3301.

[5] Y. Li , L. Li , J. H. Yu , Chem 2017, 3, 928.

[6] Y. Li , H. Yi , X. Tang , F. Li , Q. Yuan , Chem. Eng. J. 2013, 229, 50.

[7] A. Goeppert , M. Czaun , G. K. Surya Prakash , G. A. Olah , Energy Environ. Sci. 2012, 5, 7833.

[8] S. Mitchell , N. L. Michels , K. Kunze , J. Perez‐Ramirez , Nat. Chem. 2012, 4, 825. 23000996 10.1038/nchem.1403

[9] J. Jänchen , T. H. Herzog , K. Gleichmann , B. Unger , A. Brandt , G. Fischer , H. Richter , Microporous Mesoporous Mater. 2015, 207, 179.

[10] A. Zampieri , P. Colombo , G. T. P. Mabande , T. Selvam , W. Schwieger , F. Scheffler , Adv. Mater. 2004, 16, 819.

[11] S. Wang , R. Li , D. Li , Z.‐Y. Zhang , G. Liu , H. Liang , Y. Qin , J. Yu , Y. Li , J. Mater. Chem. B 2018, 6, 3254. 10.1039/c8tb00328a 32254383

[12] R. D. Zhang , K. Hedjazi , B. H. Chen , Y. X. Li , Z. G. Lei , N. Liu , Catal. Today 2016, 273, 273.

[13] F. Akhtar , L. Andersson , S. Ogunwumi , N. Hedin , L. Bergstrom , J. Eur. Ceram. Soc. 2014, 34, 1643.

[14] F. Rezaei , P. Webley , Sep. Purif. Technol. 2010, 70, 243.

[15] B. Besser , L. Haeuser , L. Butzke , S. Kroll , K. Rezwan , ACS Omega 2017, 2, 6337. 31457240 10.1021/acsomega.7b00972 PMC6645045

[16] Y.‐J. Lee , J. S. Lee , Y. S. Park , Adv. Mater. 2001, 13, 1259.

[17] X. Y. Yang , G. Tian , L. H. Chen , Y. Li , J. C. Rooke , Y. X. Wei , Z. M. Liu , Z. Deng , G. Van Tendeloo , B. L. Su , Chem. ‐ Eur. J. 2011, 17, 14987. 22113715 10.1002/chem.201101594

[18] P. Vasiliev , F. Akhtar , J. Grins , J. Mouzon , C. Andersson , J. Hedlund , L. Bergstrom , ACS Appl. Mater. Interfaces 2010, 2, 732. 20356274 10.1021/am900760w

[19] Y. Wang , Y. Tang , A. Dong , X. Wang , N. Ren , W. Shan , Z. Gao , Adv. Mater. 2002, 14, 994.

[20] H. J. Oh , M. S. Aboian , M. Y. J. Yi , J. A. Maslyn , W. S. Loo , X. Jiang , D. Y. Parkinson , M. W. Wilson , T. Moore , C. R. Yee , G. R. Robbins , F. M. Barth , J. M. DeSimone , S. W. Hetts , N. P. Balsara , ACS Cent. Sci. 2019, 5, 419. 30937369 10.1021/acscentsci.8b00700 PMC6439445

[21] M. A. Heinrich , R. Bansal , T. Lammers , Y. S. Zhang , R. Michel Schiffelers , J. Prakash , Adv. Mater. 2019, 31, 1806590. 10.1002/adma.201806590 30702785

[22] X. Zhou , Adv. Funct. Mater. 2017, 27, 1701134.

[23] J. C. Ruiz‐Morales , A. Tarancón , J. Canales‐Vázquez , J. Méndez‐Ramos , L. Hernández‐Afonso , P. Acosta‐Mora , J. R. Marín Rueda , R. Fernández‐González , Energy Environ. Sci. 2017, 10, 846.

[24] C. Minas , D. Carnelli , E. Tervoort , A. R. Studart , Adv. Mater. 2016, 28, 9993. 27677912 10.1002/adma.201603390

[25] X. Su , X. Li , C. Y. A. Ong , T. S. Herng , Y. Wang , E. Peng , J. Ding , Adv. Sci. 2019, 6, 1801670. 10.1002/advs.201801670 PMC6425437 30937261

[26] Z. Lyu , G. J. H. Lim , R. Guo , Z. Kou , T. Wang , C. Guan , J. Ding , W. Chen , J. Wang , Adv. Funct. Mater. 2018, 29, 1806658.

[27] M. Zhang , L. Li , Q. Lin , M. Tang , Y. Wu , C. Ke , J. Am. Chem. Soc. 2019, 141, 5154. 30912659 10.1021/jacs.9b01561

[28] V. Middelkoop , K. Coenen , J. Schalck , M. Van Sint Annaland , F. Gallucci , Chem. Eng. J. 2019, 357, 309.

[29] F. Magzoub , X. Li , J. Al‐Darwish , F. Rezaei , A. A. Rownaghi , Appl. Catal., B 2019, 245, 486.

[30] S. Couck , J. Lefevere , S. Mullens , L. Protasova , V. Meynen , G. Desmet , G. V. Baron , J. F. M. Denayer , Chem. Eng. J. 2017, 308, 719.

[31] X. Li , A.‐A. Alwakwak , F. Rezaei , A. A. Rownaghi , ACS Appl. Energy Mater. 2018, 1, 2740.

[32] J. Lefevere , L. Protasova , S. Mullens , V. Meynen , Mater. Des. 2017, 134, 331.

[33] H. Thakkar , S. Eastman , A. Hajari , A. A. Rownaghi , J. C. Knox , F. Rezaei , ACS Appl. Mater. Interfaces 2016, 8, 27753. 27658639 10.1021/acsami.6b09647

[34] F. Akhtar , Q. Liu , N. Hedin , L. Bergstrom , Energy Environ. Sci. 2012, 5, 7664.

[35] H. Thakkar , S. Lawson , A. A. Rownaghi , F. Rezaei , Chem. Eng. J. 2018, 348, 109.

[36] S. Couck , J. Cousin‐Saint‐Remi , S. Van der Perre , G. V. Baron , C. Minas , P. Ruch , J. F. M. Denayer , Microporous Mesoporous Mater. 2018, 255, 185.

[37] J. Lefevere , S. Mullens , V. Meynen , Chem. Eng. J. 2018, 349, 260.

[38] Y. M. Lvov , D. G. Shchukin , H. Möhwald , R. R. Price , ACS Nano 2008, 2, 814. 19206476 10.1021/nn800259q

[39] Y. Wei , P. Yuan , D. Liu , D. Losic , D. Tan , F. Chen , H. Liu , J. Zhou , P. Du , Y. Song , Chem. Commun. 2019, 55, 2110. 10.1039/c8cc10314c 30698582

[40] Y. Lvov , W. Wang , L. Zhang , R. Fakhrullin , Adv. Mater. 2016, 28, 1227. 26438998 10.1002/adma.201502341

[41] V. Abbasov , T. Mammadova , N. Aliyeva , M. Abbasov , N. Movsumov , A. Joshi , Y. Lvov , E. Abdullayev , Fuel 2016, 181, 55.

[42] J. Lin , Y. F. Luo , B. C. Zhong , D. C. Hu , Z. X. Jia , D. M. Jia , Appl. Surf. Sci. 2018, 441, 798.

[43] M. Liu , Z. Jia , D. Jia , C. Zhou , Prog. Polym. Sci. 2014, 39, 1498.

[44] C. Zhou , A. Alshameri , C. Yan , X. Qiu , H. Wang , Y. Ma , J. Porous Mater. 2013, 20, 587.

[45] J. Zhu , Y. Wang , J. Liu , Y. Zhang , Ind. Eng. Chem. Res. 2014, 53, 13711.

[46] T. Montanari , G. Busca , Vib. Spectrosc. 2008, 46, 45.

[47] D. Bahamon , L. F. Vega , Chem. Eng. J. 2016, 284, 438.

[48] T. Montanari , E. Finocchio , E. Salvatore , G. Garuti , A. Giordano , C. Pistarino , G. Busca , Energy 2011, 36, 314.

[49] F. Rezaei , P. Webley , Chem. Eng. Sci. 2009, 64, 5182.

[50] A. Aranzabal , D. Iturbe , M. Romero‐Saez , M. P. Gonzalez‐Marcos , J. R. Gonzalez‐Velasco , J. A. Gonzalez‐Marcos , Chem. Eng. J. 2010, 162, 415.

[51] B. Besser , H. A. Tajiri , G. Mikolajczyk , J. Mollmer , T. C. Schumacher , S. Odenbach , R. Glaser , S. Kroll , K. Rezwan , ACS Appl. Mater. Interfaces 2016, 8, 3277. 26760054 10.1021/acsami.5b11120

[52] A. Ojuva , F. Akhtar , A. P. Tomsia , L. Bergstrom , ACS Appl. Mater. Interfaces 2013, 5, 2669. 23484540 10.1021/am400122r

[53] F. Akhtarw , L. Bergstrom , J. Am. Ceram. Soc. 2011, 94, 92.

[54] A. Khelifa , L. Benchehida , Z. Derriche , J. Colloid Interface Sci. 2004, 278, 9. 15313632 10.1016/j.jcis.2004.05.033

[55] J. A. Dunne , M. Rao , S. Sircar , R. J. Gorte , A. L. Myers , Langmuir 1996, 12, 5896.

