# Supplementary material for: Fabricating Mechanically Robust Binder‐Free Structured Zeolites by 3D Printing Coupled with Zeolite Soldering: A Superior Configuration for CO2 Capture
Source: Adv Sci (Weinh). 2019 Jul 1;6(17):1901317. doi: 10.1002/advs.201901317 (PMC6724348; doi:10.1002/advs.201901317)
Supplement: Supplementary file 1 — Supplementary [file ADVS-6-1901317-s001.pdf]

Copyright WILEY-VCH Verlag GmbH & Co. KGaA, 69469 Weinheim, Germany, 2019.

ADVANCED SCIENCE
Open Access

Supporting Information

for *Adv. Sci.,* DOI: 10.1002/advs.201901317

Fabricating Mechanically Robust Binder-Free Structured Zeolites by 3D Printing Coupled with Zeolite Soldering: A Superior Configuration for CO_2 Capture

Shuang Wang, Pu Bai, Mingzhe Sun, Wei Liu, Dongdong Li, Wenzheng Wu, Wenfu Yan, Jin Shang, and Jihong Yu**

Copyright WILEY-VCH Verlag GmbH & Co. KGaA, 69469 Weinheim, Germany, 2019.

Supporting Information

Fabricating Mechanically Robust Binder-Free Structured Zeolites by 3D Printing Coupled with Zeolite Soldering: A Superior Configuration for CO_2 Capture

Shuang Wang, Pu Bai, Mingzhe Sun, Wei Liu, Dongdong Li, Wenzheng Wu, Wenfu Yan, Jin Shang, Jihong Yu**

S. Wang, P. Bai, Prof. W. Yan, Prof. J. Yu
State Key Laboratory of Inorganic Synthesis and Preparative Chemistry, College of Chemistry, Jilin University, Changchun 130012, China
E-mail: jihong@jlu.edu.cn
M. Sun, Dr. J. Shang
School of Energy and Environment, City University of Hong Kong, Tat Chee Ave, Kowloon, Hong Kong, China
W. Liu, Dr. W. Wu
School of Mechanical and Aerospace Engineering, Jilin University, Changchun 130025, China
Dr. D. Li
Key Laboratory of Automobile Materials of MOE, Department of Materials Science and Engineering, Jilin University, Changchun 130012, China
Prof. J. Yu
International Center of Future Science, Jilin University, Changchun 130012, China

Experimental section

***Preparation of 3D-printed zeolite monoliths*:** To make a desired printing ink, the mixtures were prepared with NaX zeolite powders (SiO_2/Al_2O_3 = 2.37, the average particle size 2-3 μm, Luoyang Jalon Micro-nano New Materials Co., Ltd.), halloysite nanotubes (HNTs, SiO_2/Al_2O_3 = 2.02, Shanxi Province, China) as the main printing ink additives, colloidal silica (LudoxAS-40, Sigma-Aldrich) as extra silicon source, and hydroxypropyl methylcellulose (HPMC, Aladdin) as a plasticizing agent. More specifically, NaX powders and HNTs were mixed rigorously by an agate mortar and a mechanical mixer (Eumix R30, Fluko Shanghai, China), resulting in homogeneous powder mixture ‘Part 1’. Meanwhile, the colloidal silica and HPMC were added into DI water to get a homogeneous mixture ‘Part 2’. In the next step, both mixtures were mixed and stirred for 1-2 h. After sonication, a printing ink with optimal viscosity and homogeneity was obtained, which contained x wt% NaX zeolites, y wt% HNTs (x+y=97, y=0, 7, 14, 21), 1 wt% silica (added in the form of colloidal silica), and 2 wt% HPMC (except for water). The solid content of printing inks was 45±1 wt%. The printing inks were then transferred to a 10 cm^3 syringe (Shanghai Yichang Instrument Market Management Co., Ltd., China) carefully. The inks were printed through a nozzle in a pneumatic-injection 3D printing system (EV-3 system, USA) followed by a movement controlled by computer in x, y, and z direction. The mixing and printing temperature was kept at 20-25 °C. After printing, the monoliths were freeze-dried on a vacuum freeze dryer (LGJ-10E, Four-Ring Science Instrument Plant Beijing Co. Ltd., China) for 24 h under vacuum condition to prevent collapse and cracking, and the cold trap temperature was set at -60 °C. Then the monoliths were calcined with a heating rate of 5 °C/min and kept at 650 °C for 2 h. The obtained 3D-printed zeolite monoliths with binder (HNTs) were denoted as ZM-WB. The overall size of the ZM-WB as well as wall thickness and channel size can be easily controlled by the nozzle size and the computer program. For the representative cuboid honeycomb structure (20 mm

length, 20 mm width, and 10 mm height) was used for studies, the rod diameter and rod spacing were 1 mm. This corresponds to an open porosity of 50% and channels per square inch (cpsi) of 161. Considering that the HNTs content has a significant influence on the rheological properties and extrusion abilities of printing inks as well as the physical and chemical properties of the final configuration, the printing ink with a 14 wt% HNTs was used (the formulation of printing inks was 83 wt% NaX zeolites, 14 wt% HNTs , 1 wt% silica, and 2 wt% HPMC) in the following studies.

Preparation of 3D-printed binder-free zeolite monoliths**:** The preparation of binder-free zeolite monoliths were carried out by a hydrothermal crystallization treatment. Firstly, 3 M sodium hydroxide (Sinopharm chemical reagent of Shanghai, China) solution was prepared. Secondly, a mixture was obtained by adding 10 g of ZM-WB into a teflon-lined stainless steel autoclave with 25 mL of 3 M sodium hydroxide solution and then crystallized at 95 °C for 8 h under autogenous pressure. The final molar composition of the synthesis gel was $6.2Na_2O$: $2.3SiO_2$: Al_2O_3: $230H_2O$. During hydrothermal crystallization, HNTs in ZM-WB acted as the main silicon source and the aluminum source and colloidal silica as the extra silicon source, respectively. After the hydrothermal crystallization, the obtained zeolite monoliths were washed thoroughly until the pH value of the washed water was below 9, dried in an oven at 80 °C for 24 h, and then calcined with a heating rate of 5 °C/min and kept at 550 °C for 1 h. The resultant 3D-printed binder-free zeolite monoliths were denoted as ZM-BF.

Rheological measurements

Rheological measurements were applied to determine the viscosity of the printing inks with different HNTs concentration (the content of HNTs is 0, 7, 14, and 21 wt%) as a function of the shear rate (Discovery HR-2 hybrid rheometer, TA instrument, New Castle, DE, USA). Shear rates between 0.01 and 10 s^{-1} were applied at a constant temperature of 25 °C.

Mechanical strength measurement

For comparison, five types of commercial binders: colloidal silica (LudoxAS-40, Sigma-Aldrich), boehmite (Aluminum Corporation of China Limited), attapulgite (Anhui Mingmei MinChem Co., Ltd.), bentonite (Sinopharm Chemical Reagent Co., Ltd.), and kaolin (Sinopharm Chemical Reagent Co., Ltd.) were chosen as printing ink additives to replace HNTs for fabricating zeolite monoliths, while other experimental parameters including the formulations of printing ink additives and solid content retained the same. The length, width, and height of all 3D-printed monoliths used during the experiment were 2 cm, 2 cm, and 1 cm, respectively. The nozzle diameter was kept at 0.90 mm and the off-set in z-direction was set to 0.5 mm. The zeolite monoliths printed with various printing ink additives were denoted as ZM@bentonite, ZM@attapulgite, ZM@colloidal silica, ZM@boehmite, and ZM@kaolin, respectivly. The compressive strengths of zeolite monoliths printed with different binders were measured by an electronic universal testing machine (UTM6104, Shenzhen Suns Co. Ltd., China) with a 10 kN measuring cell at 0.4 mm/min. The average compressive strength was calculated from at least three independent tests.

Characterization

Arigaku D/MAX 2500/PC X-ray diffractometer with CuKα radiation (λ = 1.5418 Å) was used to collect Powder X-ray diffraction (XRD) data. The morphologies of samples were characterized by scanning electron microscopy (SEM, JSM-7800F, JSM-6700F, Japan) and transmission electron microscopy (TEM, Tecnai G2 S-Twin F20). X-ray fluorescence (XRF, AXIOS, Holand PANalytical Co., Ltd) was used for analyzing the elemental composition of specimens. Nitrogen adsorption-desorption isotherms at liquid N_2 temperature were carried out with a Micromeritrics ASAP 2020 instrument. Before measurement, all the samples were degassed at 350 °C for 12 h. The textural properties including total surface area, external surface area, and micropore volume were measured using Brunauer-Emmett-Teller (BET) equation and t-plot method, respectively. The total pore volume was obtained from the

capacity of N_2 adsorbed at P/P_0 = 0.99. The pore size distributions (PSDs) of synthesized samples were determined by the density functional theory (DFT) method. The macropore volume and the PSDs were determined by mercury intrusion porosimetry (Micromeritics Auto Pore III 9410).

Adsorption isotherms of N_2, CH_4, and CO_2

Single component equilibrium adsorption isotherms for N_2, CH_4, and CO_2 were performed on Micrometrics ASAP 2020 instrument. Top-commercial CO_2 sorbents including NaX extrudates (d = 1.5 mm, C-NaX-1, UOP), NaX pellets (d = 1.6-2.5 mm, C-NaX-2, UOP), NaX extrudates (d = 3 mm, C-NaX-3, UOP), and granular activated carbon (d = 1-2 mm, GAC, Tianjin Fuchen Chemical Reagents Company) were employed in this work for comparison. Prior to each adsorption experiment, all the samples were degassed at 350 ^{o}C for 12 h under vacuum to desorb all adsorbed gases and water.

Separation factor (α)

Using the pure component isotherms, the adsorption selectivity is given as

$$\text{Selectivity} = \frac{q_1/q_2}{p_1/p_2}$$

where $q_{1,2}$ and $p_{1,2}$ are the uptake and the partial pressure of components 1 and 2, respectively.

Column breakthrough experiments

The breakthrough experiments were carried out on a lab made rig. An experimental set-up for the dynamic column CO_2 separation is shown in Schematic S1. In order to meet the size of the packed bed adsorber, ZM-WB and ZM-BF were printed into cylindrical monolithic adsorbents with a diameter and height of 10 mm. The nozzle diameter during printing process was kept at 0.51 mm and the off-set in z-direction was set to 0.4 mm. Commercial NaX zeolites mentioned above were employed for comparison. Different adsorbents were loaded in the column (length 310 mm, I.D = 10 mm, volume = 24.4 cm^3) occupying the bed height of

90 mm in the middle of column. The remaining column volume was filled by the glass beads of 3 mm diameter previously washed and then dried overnight at 130 ^oC. The sample was further degassed under inert gas (Ar) flowing at the rate of 20 mL/min and 350 ^oC for 4.5 hours to ensure the complete degassing. The temperature ramping rate for attaining the degassing temperature was 10 ^oC/min. After the degassing, the adsorbents were cooled down to room temperature under Argon flow. The breakthrough experiments were then carried out at room temperature (23 ± 1 ^oC) and atmospheric pressure and the mixed gas flow rate was 50 mL/min. The concentration of the gases coming out of the column was monitored using an online mass spectrometry-based analyzer (Stanford Research Systems UGA300) till the outlet concentration became almost equal to gas inlet concentration. The adsorbents were then regenerated again at 350 ^oC under argon flow for 3.5 hours for subsequent breakthrough experiments. The breakthrough capacities were corrected for gas analyzer response and dead space time by subtracting the blank capacity carried under similar conditions with only difference that the column was fully packed with glass beads only. The specific breakthrough capacities of adsorbent column for different gases and specific breakthrough capacities of the column were calculated as

$$\chi_{BT} = \frac{F_r}{22.4 * w}\left(\int_0^{t_s}(C_0 - C)dt - \int_0^{t_b}(C_0 - C)dt\right)$$

Where χ_{BT} is specific breakthrough capacity of adsorbent in mmol/g.

t_s is breakthrough time (at $C = 0.05C_0$) in minutes when sample is loaded in column.

t_b is breakthrough time (at $C = 0.05C_0$) in minutes for blank column.

F_r is total flow rate of the gas component in mL/min.

w is weight of adsorbent after activation in gram.

C_0 is concentration of component in feed.

C is outlet concentration at time t.

Schematic S1 shows a scheme of the setup used for the column breakthrough experiments. Different gas mixtures can be prepared by a set of mass flow controllers.

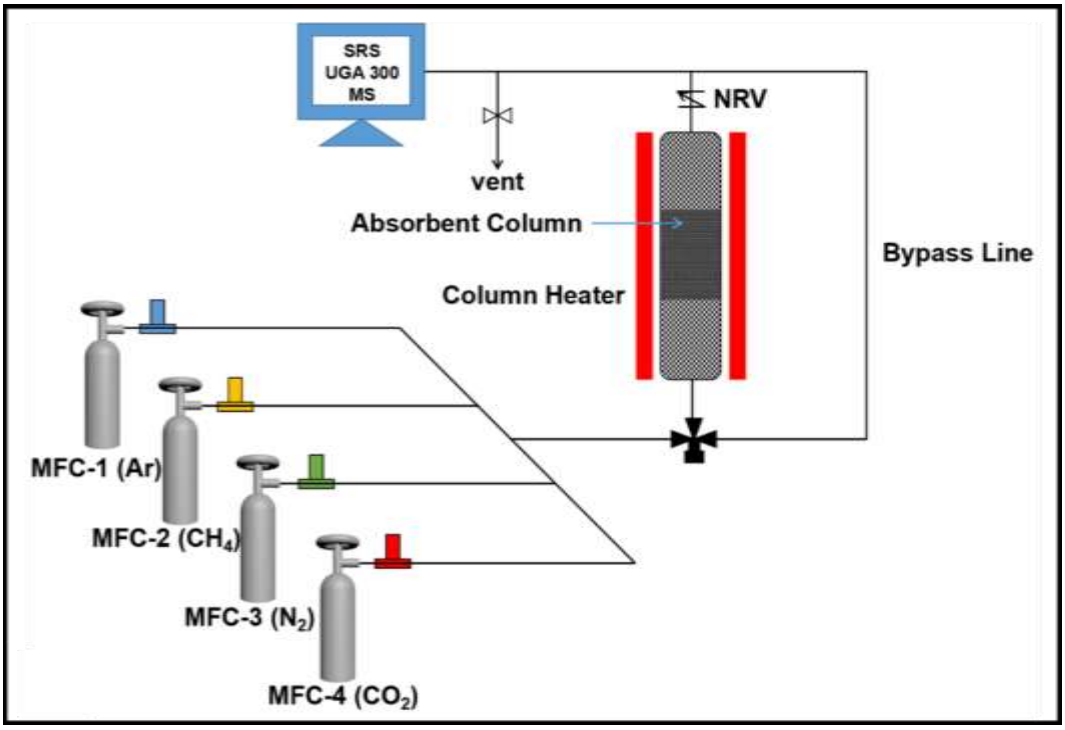

Schematic S1. Scheme of a set-up for the dynamic column CO_2 separation.

Table S1. Geometric parameters of ZM-BF with different geometries shown in Figure 2a.

Samples	Overall size (mm)	Rod diameter (mm)	Rod Spacing (mm)	Open porosity[e] (%)	cpsi[f]
S1	Cuboid<br>L[a]=20, W[b]=20, H[c]=10	1.00	1.00	50	161
S2	Cylinder<br>D[d]=20, H=3	0.58	0.48	45	616
S3	Cylinder<br>D=20, H=10	0.77	1.25	62	156
S4	Cuboid<br>L=15, W=15, H=10	0.36	0.68	65	645
S5	Cylinder<br>D=20, H=8	0.59	0.48	45	616

[a]Length of cuboid structure.

[b]Width of cuboid structure.

[c]Height of cuboid structure.

[d]Diameter of cylinder structure.

[e]Open porosity is calculated based on the rod diameter and rod spacing.

[f]Channels per square inch (cpsi).

Table S2. Textural properties of the parental NaX powders, HNTs, ZM-WB, and ZM-BF.

**Samples**	**$^{a}S_{BET}$ (m^2/g)**	**$^{b}S_{micro}$ (m^2/g)**	**$^{b}S_{ext}$ (m^2/g)**	**$^{b}V_{micro}$ (cm^3/g)**	**$^{c}V_{mes}$ (cm^3/g)**
NaX powders	695	663	32	0.317	0.017
HNTs	62	3.5	58.5	0.001	0.216
ZM-WB	517	470	47	0.242	0.090
ZM-BF	611	571	40	0.295	0.040

$^{a}S_{BET}$ (total surface area) calculated by applying the BET equation using the linear part ($0.05 < P/P_0 < 0.30$) of the adsorption isotherm. $^{b}S_{micro}$ (micropore area), $^{b}S_{ext}$ (external surface), and $^{b}V_{micro}$ (micropore volume) calculated using the t-plot method. $^{c}V_{meso}$ (mesopore volume) calculated using the BJH method (from desorption).

Table S3. Mechanical testing data for 3D-printed zeolite monoliths fabricated with various printing ink additives.

Samples	ZM@ boehmite	ZM@colloidal silica	ZM@ attapulgite	ZM@ bentonite	ZM@ kaolin	ZM-WB	ZM-BF
Compressive strength (MPa)[a]	0.11	0.31	0.62	1.28	1.89	4.32	5.24
Young's modulus (MPa)[b]	2.89	5.49	17.5	22.4	32.5	29.4	42.8
Fracture strain (%)[c]	4.15	5.67	2.55	6.28	5.89	12.28	11.40

[a] Compressive strength is the maximum stress value before fracture.

[b] The Young's modulus is calculated from the slope of stress–strain curves before fracture.

[c] The fracture strain is a parameter related to the load bearing capability and ductility. The larger the value is, the better ductility of the sample will be.

Table S4. Comparisons of mechanical strength of self-supporting structured zeolites fabricated via various methods.

Adsorbents	Fabricating methods	Mechanical strength (MPa)	References
Zeolite NaKA	Pulsed current processing	1.6-2.2	[1]
Zeolite 13X	Freeze gelation	0.42-1.38	[2]
Zeolite-geopolymer composite	Gel casting	3	[3,4]
Zeolite 13X	Freeze casting and sacrificial templating	0.045 – 0.146	[5]
Zeolite 13X	Freeze casting	0.054 - 0.69	[6]
Zeolite 13X	Slip casting	0.207 -0.748	[7]
H-ZSM-5 zeolite	Direct extrusion technique	2.58 ± 0.5	[8]
Zeolite silicalite-1	Sacrificial templating	0.085	[9]
Zeolite ZSM-5	3D printing	0.22-1.54	[10]
Zeolite 13X	3D printing	0.3-0.69	[11]
Zeolite 5A	3D printing	0.05-0.53	[11]
Torlon@zeolite 13X composite	3D printing	210	[12]
Torlon@zeolite 5A composite	3D printing	210	[12]
ZM@boehmite	3D printing	0.11	
ZM@colloidal silica	3D printing	0.31	
ZM@attapulgite	3D printing	0.61	
ZM@bentonite	3D printing	1.28	This work
ZM@kaolin	3D printing	1.89	
ZM-WB	3D printing	4.32	
ZM-BF	3D printing	5.24	

Not: the length, width, and height of the printed zeolite monoliths with various printing ink additives in this work were 2 cm, 2 cm, and 1 cm, respectively, while the nozzle diameter was kept at 0.90 mm and the off-set in z-direction was set to 0.5 mm.

Table S5. Comparisons of equilibrium CO_2 uptake on various structured adsorbents.

	Adsorbent materials	Adsorbing capacity of CO_2 (mmol/g)*	Test conditions	References
Porous carbon	Carbon monoliths	1.6	303 K, 1 bar	[13]
	Activated carbon-zeolite NaUSY monolith	3.25	293 K, 1 bar	[14]
	Porous carbon monoliths	3.10	298 K, 1 bar	[15]
	Carbon fibre composite monolith	2.71	298 K, 1 bar	[16]
	Nitrogen-Doped Porous Carbon Monolith	3.13	298 K, 1 bar	[17]
	ZSM-5/carbon honeycomb monolith	2.2	298 K, 1 bar	[18]
MOFs	MOFs($Cu_3(BTC)_2$)-Carbon Monoliths	2.75	298 K, 1 bar	[19]
	MOF-74(Ni) monolith	4.0	298 K, 1 bar	[20]
	UTSA-16(Co) monolith	3.0	298 K, 1 bar	[20]
	UTSA-16@Co-kaolin	3.0	298 K, 1 bar	[21]
	MOF-74 coated cordierite monolith	1.7	298 K, 1 bar	[22]
	UTSA-16 coated cordierite monolith	1.1	298 K, 1 bar	[22]
Aminosilica	Tetraethylenepentamine-silica monolith	2.23	298 K, 1 bar	[23]
	polyethylenimine-silica monoliths	1.4	298 K, 1 bar	[24]
	Trimethoxysilane@SBA-15 monolith	1.51	301 K, 1 bar	[25]
Zeolite	Binder-free zeolite NaKA monolith	4.2	298 K, 1 bar	[1]
	Zeolite NaX monolith	5	303 K, 1 bar	[2]
	NaX monolith	4.5	298 K, 1 bar	[11]
	5A monolith	4.4	298 K, 1 bar	[11]
	Torlon-NaX -monolith	1.83	308 K, 1 bar	[12]
	Torlon-5A-monolith	1.51	308 K, 1 bar	[12]
	ZSM-5 monolith	1.4	302 K, 1 bar	[26]
	SAPO-34 monolith	2.7	303 K, 1 bar	[27]
	Self-supporting 5A zeolite monolith	3.8	273 K, 1 bar	[28]
	ZM-WB (Zeolite NaX)	4.49	298 K, 1 bar	This work
	ZM-BF (Zeolite NaX)	5.58	298 K, 1 bar	
	ZM-BF	6.55	273 K, 1 bar	

* CO_2 uptake obtained from single component equilibrium adsorption isotherms.

Table S6. Comparison of the equilibrium CO_2 adsorption performance of ZM-BF with the parental NaX powders, ZM-WB, and various commercial benchmark NaX zeolites used in this study.

**Adsorbents**	**CO_2 uptake (mmol/g)[a]**				**Separation factor at 1 bar**		
	0.02 bar	0.15 bar	0.5 bar	1 bar	CO_2/CH_4[b] (2/98, v/v)	CO_2/N_2[c] (15/85, v/v)	CO_2/CH_4[d] (50/50, v/v)
ZM-BF	2.53	4.20	4.96	5.58	193.6	66.49	14.3
NaX powders	2.38	4.16	5.01	5.68	175.3	63.54	14.0
ZM-WB	1.79	3.21	3.92	4.49	174.0	63.44	14.7
C-NaX-1	1.72	3.29	4.21	4.72	NM		
C-NaX-2	1.52	3.02	3.91	4.34			
C-NaX-3	1.78	3.24	4.09	4.49			
GAC	0.118	0.472	0.875	1.20			

[a] CO_2 uptake obtained from single component equilibrium adsorption isotherms.

[b] The value calculated from CO_2 uptake at 0.02 bar/CH_4 uptake at 0.98 bar.

[c] The value calculated from CO_2 uptake at 0.15 bar/N_2 uptake at 0.85 bar.

[d] The value calculated from CO_2 uptake at 0.5 bar/CH_4 uptake at 0.5 bar.

Not measured (NM).

Table S7. SiO_2/Al_2O_3 ratios of NaX zeolite powders, HNTs, ZM-WB, and ZM-BF.

Samples	SiO_2/Al_2O_3[a]
HNT_S	2.02
NaX powders	2.37
ZM-WB	2.32
ZM-BF	2.26

[a] Measured by using an X-ray fluorescence analyzer (XRF).

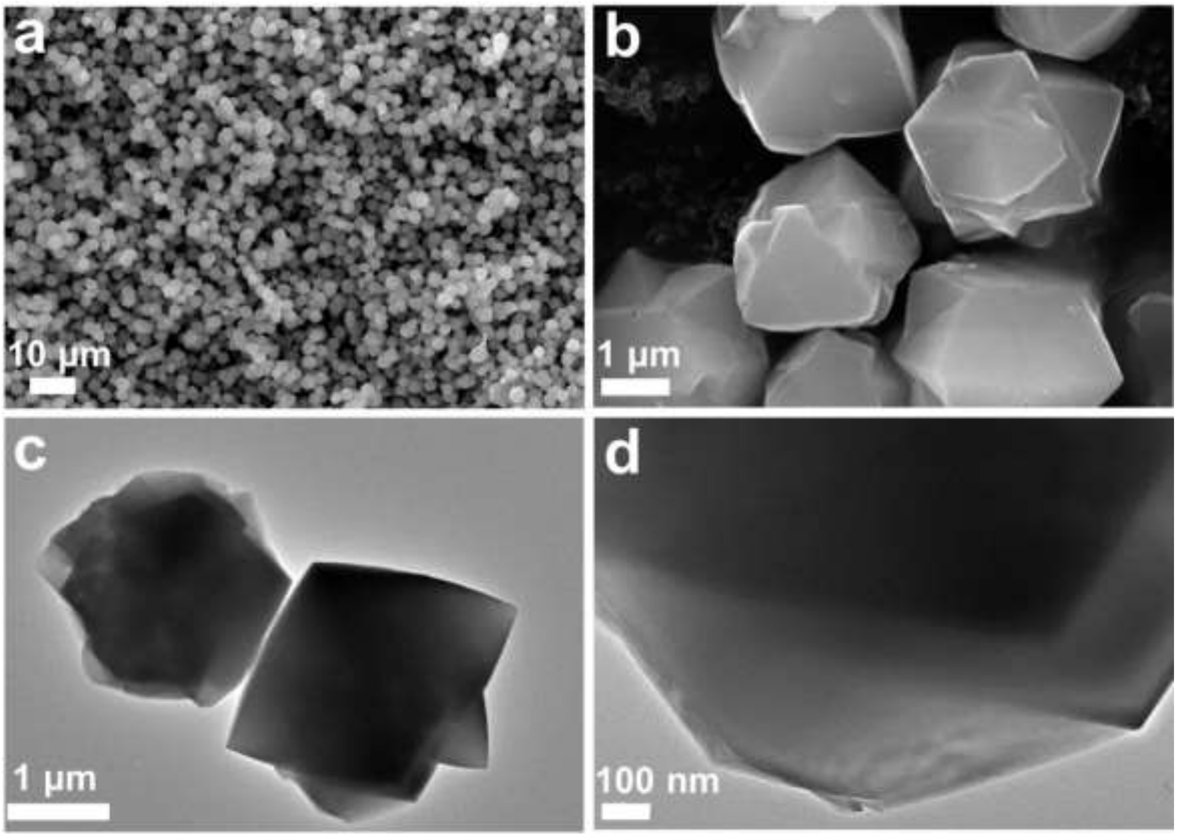

Figure S1. SEM (a,b) and TEM (c,d) images of the parental zeolite NaX powders.

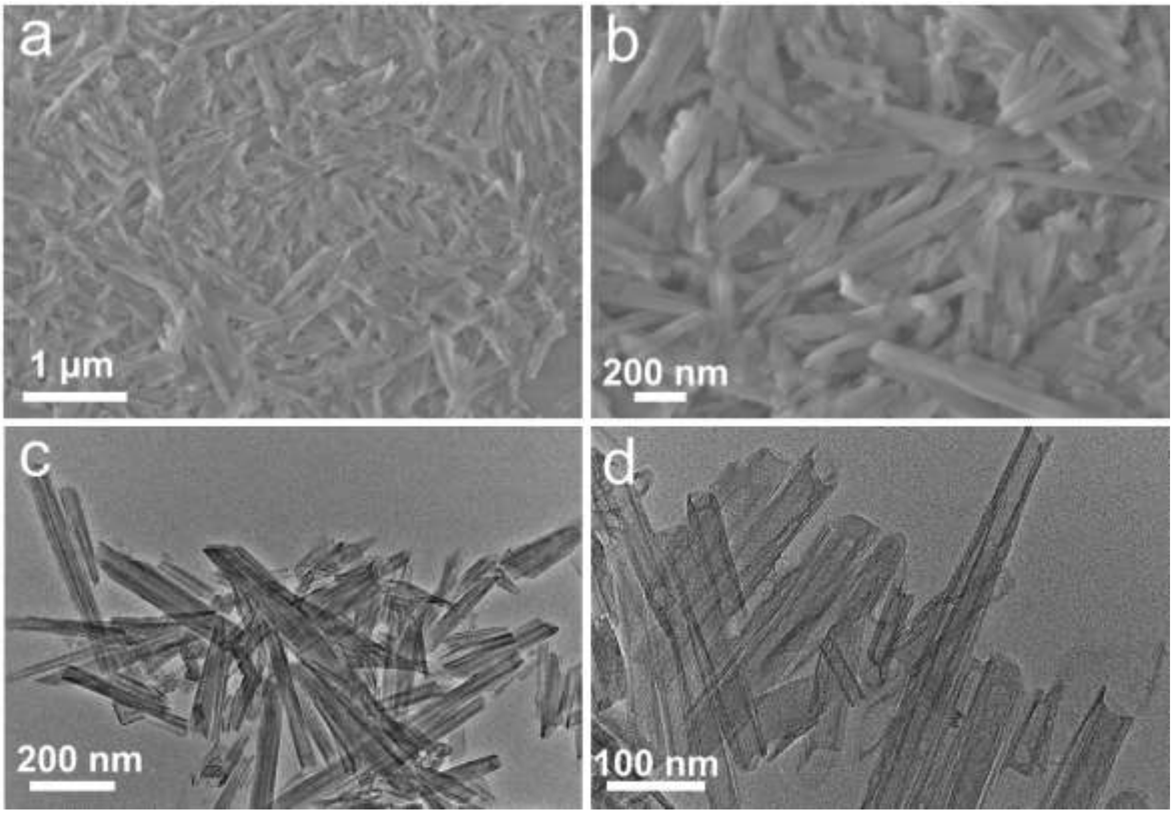

Figure S2. SEM (a,b) and TEM (c,d) images of HNTs with typical hollow nanotube structure.

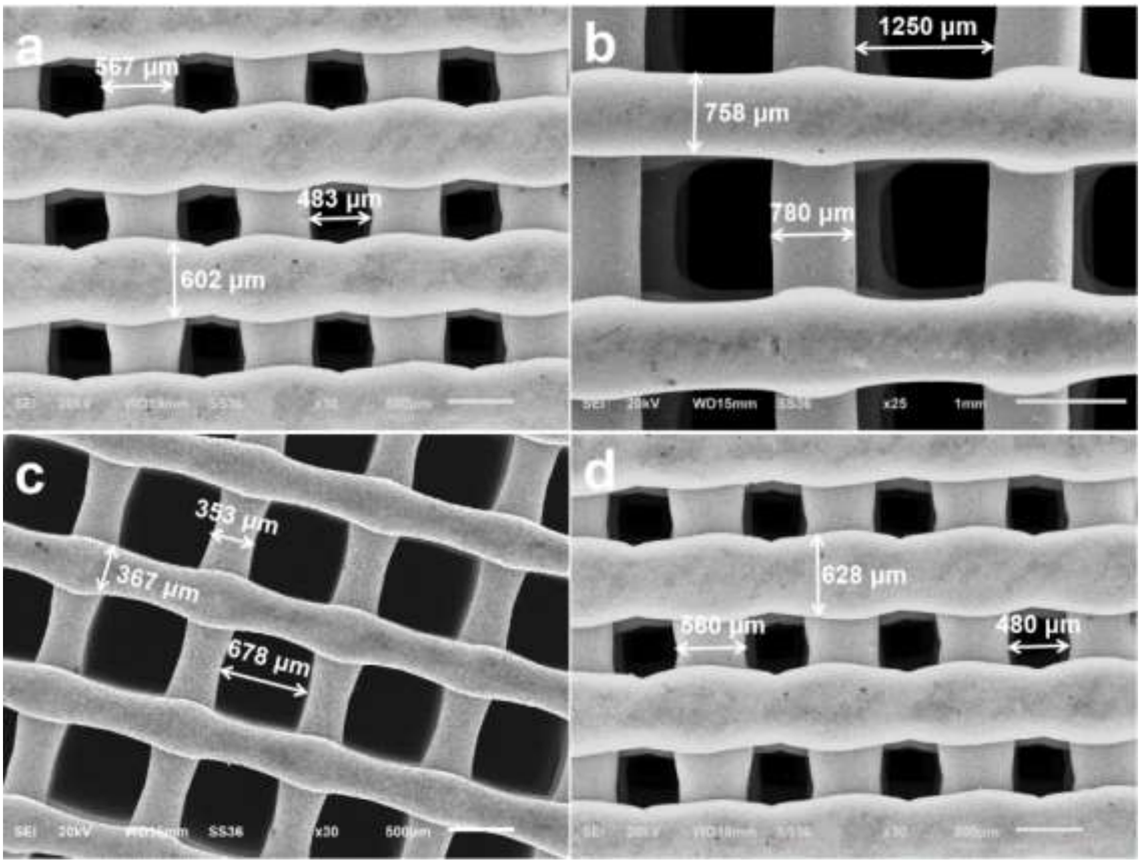

Figure S3. Low-magnification SEM images of the top view of 3D-printed zeolite monoliths with tailorable geometries in Figure 2a: (a) S2, (b) S3, (c) S4; (d) S5.

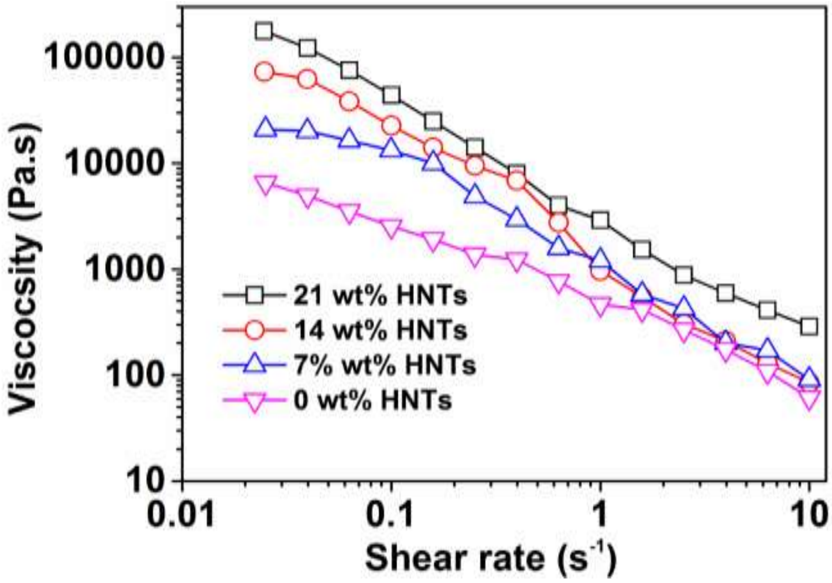

Figure S4. The rheological properties of printing inks with different HNTs contents.

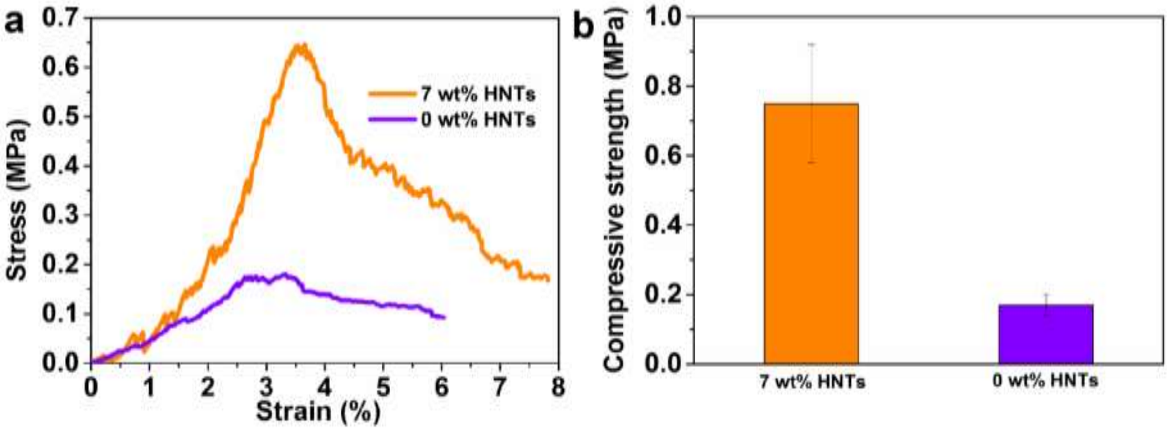

Figure S5. Representative stress-strain curves (a) and compressive strength (b) of 3D-printed zeolite monoliths with different varying HNTs contents.

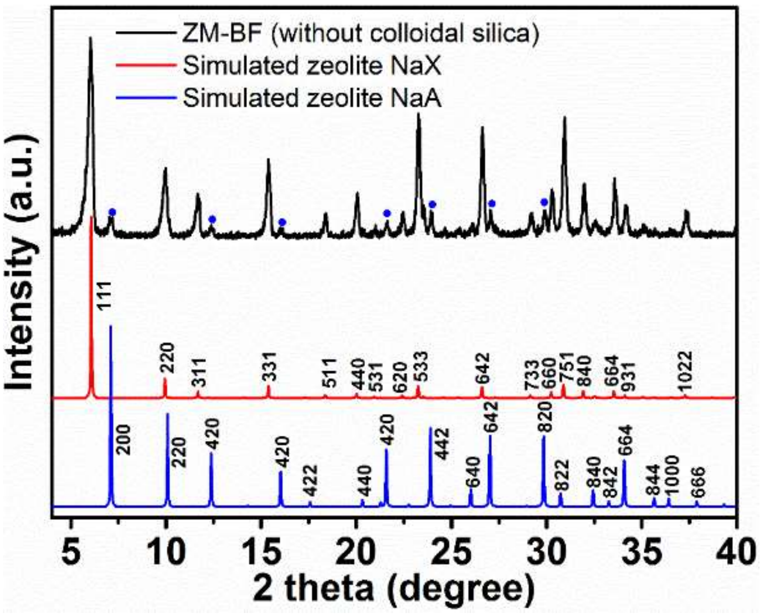

Figure S6. PXRD pattern of ZM-BF without adding colloidal silica in the printing inks.

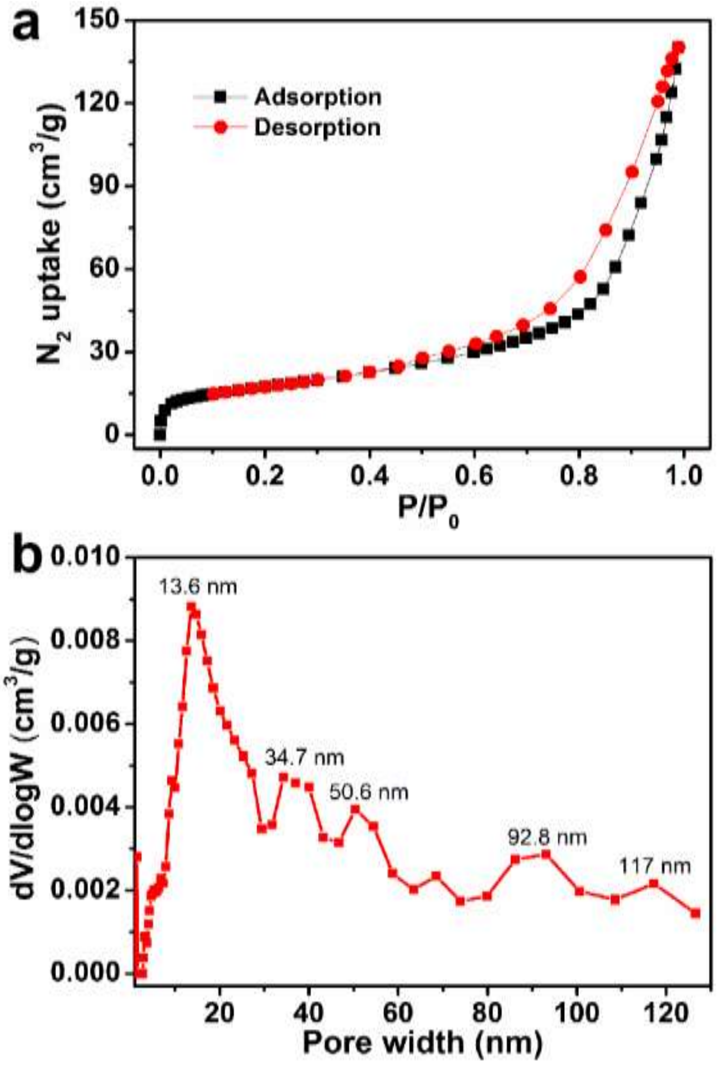

Figure S7. (a) N_2 adsorption-desorption isotherm of HNTs; (b) the pore size distribution curve of HNTs derived from density functional theory (DFT) method.

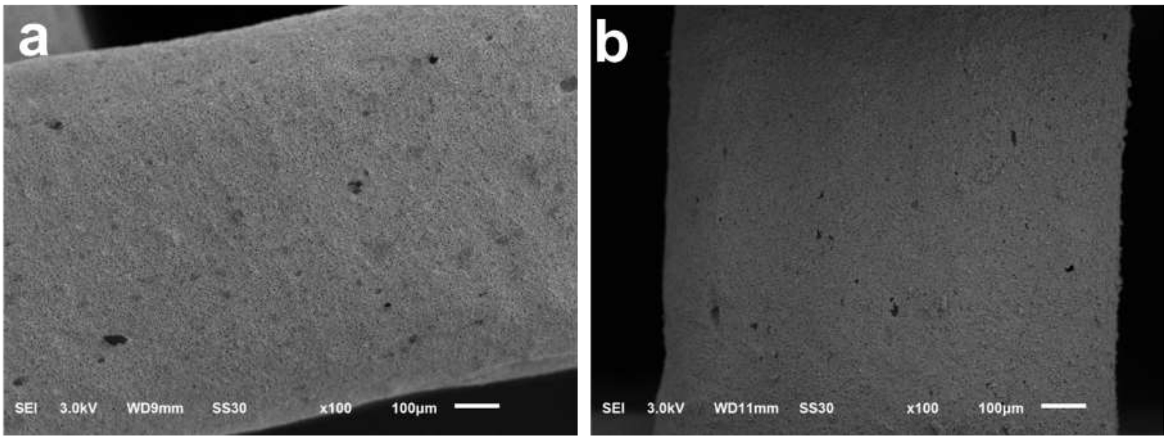

Figure S8. SEM images of ZM-WB (a) and ZM-BF (b).

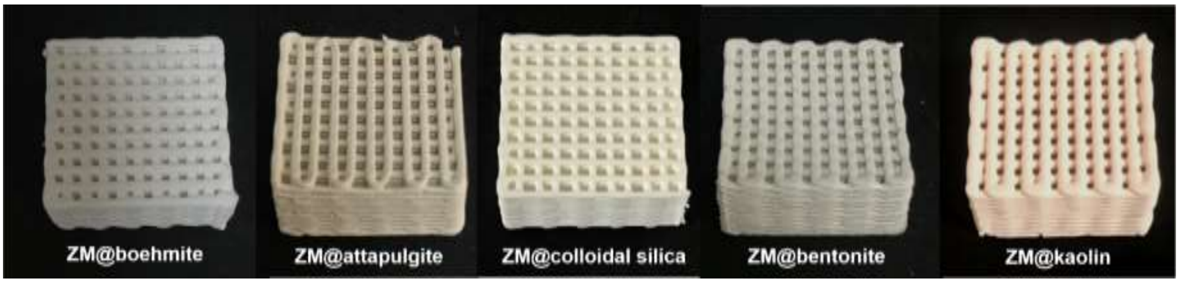

Figure S9. 3D-printed zeolite monoliths by using five types of commercial binders (boehmite, attapulgite, colloidal silica, bentonite, and kaolin) as printing ink additives.

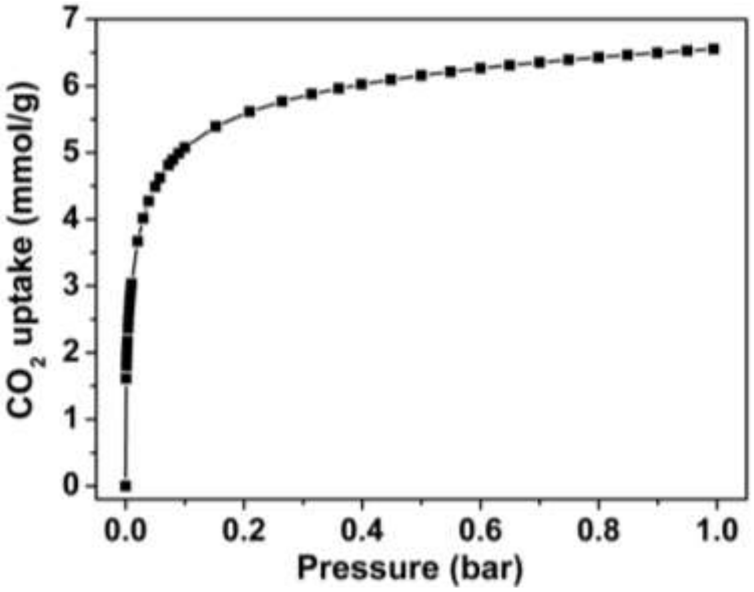

Figure S10. CO_2 adsorption isotherm of ZM-BF under 273 K and 1 bar.

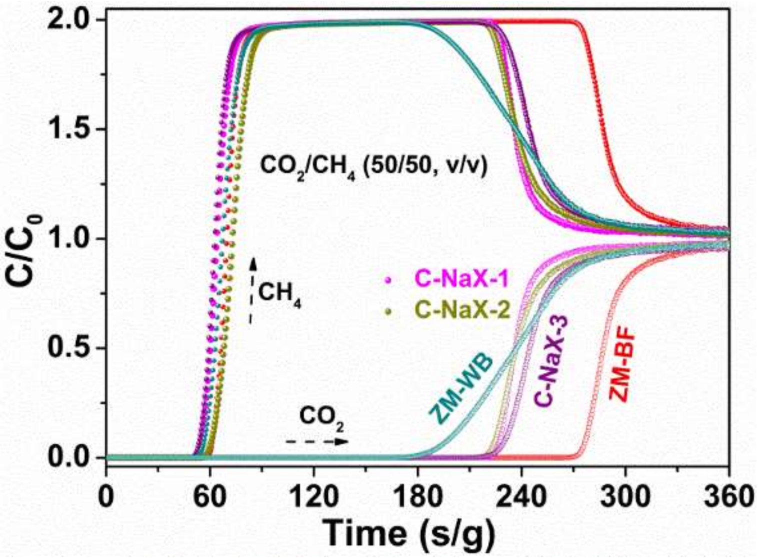

Figure S11. The column breakthrough curves for CO_2/CH_4 (50/50, v/v) over ZM-WB and ZM-BF, and other commercial CO_2 adsorbents.

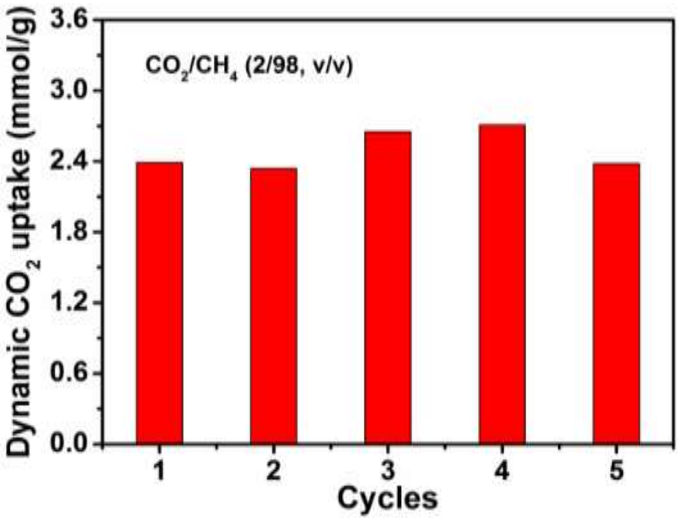

Figure S12. The recyclability of ZM-BF under CO_2/CH_4: 2/98 binary gas system at room temperature and 1 bar.

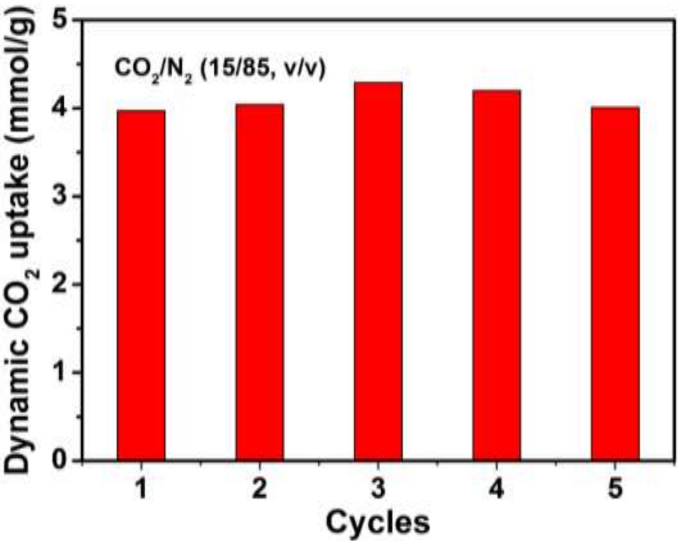

Figure S13. The recyclability of ZM-BF under CO_2/N_2: 15/85 binary gas system at room temperature and 1 bar.

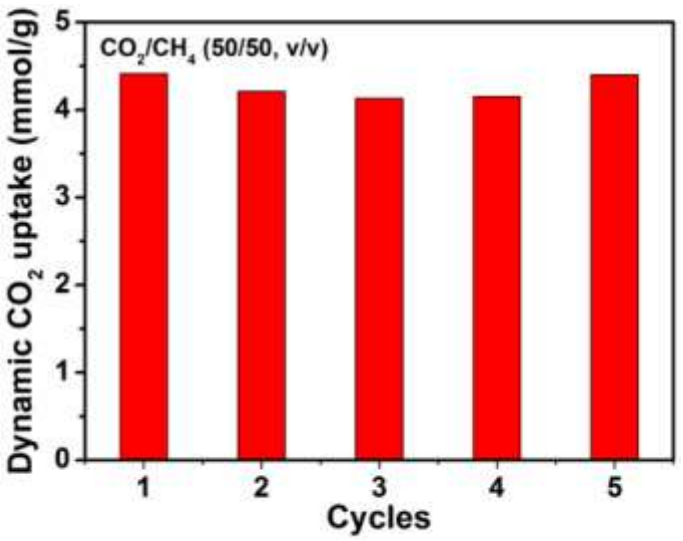

Figure S14. The recyclability of ZM-BF under CO_2/CH_4: 50/50 binary gas system at room temperature and 1 bar.

References

[1] F. Akhtar, Q. Liu, N. Hedin, L. Bergstrom, *Energy Environ. Sci.* **2012**, *5*, 7664.
[2] B. Besser, L. Haeuser, L. Butzke, S. Kroll, K. Rezwan, *ACS Omega* **2017**, *2*, 6337.
[3] E. Papa, V. Medri, S. Amari, J. Manaud, P. Benito, A. Vaccari, E. Landi, *J. Cleaner Prod.* **2018**, *171*, 76.
[4] M. Minelli, E. Papa, V. Medri, F. Miccio, P. Benito, F. Doghieri, E. Landi, *Chem. Eng. J.* **2018**, *341*, 505.
[5] B. Besser, H. A. Tajiri, G. Mikolajczyk, J. Mollmer, T. C. Schumacher, S. Odenbach, R. Glaser, S. Kroll, K. Rezwan, *ACS Appl. Mater. Interfaces* **2016**, *8*, 3277.
[6] A. Ojuva, F. Akhtar, A. P. Tomsia, L. Bergstrom, *ACS Appl. Mater. Interfaces* **2013**, *5*, 2669.
[7] F. Akhtarw, L. Bergstrom, *J. Am. Ceram. Soc.* **2011**, *94*, 92.
[8] A. Aranzabal, D. Iturbe, M. Romero-Saez, M. P. Gonzalez-Marcos, J. R. Gonzalez-Velasco, J. A. Gonzalez-Marcos, *Chem. Eng. J.* **2010**, *162*, 415.
[9] Yun-Jo Lee, Jin Seok Lee, Yong Soo Park, K. B. Yoon, *Adv. Mater.* **2001**, *13*, 1259.
[10] J. Lefevere, L. Protasova, S. Mullens, V. Meynen, *Mater. Des.* **2017**, *134*, 331.
[11] H. Thakkar, S. Eastman, A. Hajari, A. A. Rownaghi, J. C. Knox, F. Rezaei, *ACS Appl. Mater. Interfaces* **2016**, *8*, 27753.
[12] H. Thakkar, S. Lawson, A. A. Rownaghi, F. Rezaei, *Chem. Eng. J.* **2018**, *348*, 109.
[13] J. Singh, H. Bhunia, S. Basu, *J. Ind. Eng. Chem.* **2018**, *60*, 321.
[14] Q. Zhao, F. Wu, K. Xie, R. Singh, J. Zhao, P. Xiao, P. A. Webley, *Chem. Eng. J.* **2018**, *336*, 659.
[15] A. C. Juhl, C.-P. Elverfeldt, F. Hoffmann, M. Froeba, *Microporous Mesoporous Mater.* **2018**, *255*, 271.
[16] R. Thiruvenkatachari, S. Su, X. X. Yu, Y. Jin, *Int. J. Greenhouse Gas Control* **2015**, *42*, 415.
[17] G.-P. Hao, W.-C. Li, D. Qian, A.-H. Lu, *Adv. Mater.* **2010**, *22*, 853.
[18] A. Masala, J. G. Vitillo, G. Mondino, G. Martra, R. Blom, C. A. Grande, S. Bordiga, *Ind. Eng. Chem. Res.* **2017**, *56*, 8485.
[19] D. Qian, C. Lei, G.-P. Hao, W.-C. Li, A.-H. Lu, *ACS Appl. Mater. Interfaces* **2012**, *4*, 6125.
[20] H. Thakkar, S. Eastman, Q. Al-Naddaf, A. A. Rownaghi, F. Rezaei, *ACS Appl. Mater. Interfaces* **2017**, *9*, 35908.
[21] S. Lawson, Q. Al-Naddaf, A. Krishnamurthy, M. S. Amour, C. Griffin, A. A. Rownaghi, J. C. Knox, F. Rezaei, *ACS Appl. Mater. Interfaces* **2018**, *10*, 19076.
[22] F. Rezaei, S. Lawson, H. Hosseini, H. Thakkar, A. Hajari, S. Monjezi, A. A. Rownaghi, *Chem. Eng. J.* **2017**, *313*, 1346.
[23] H. Thakkar, S. Eastman, A. Al-Mamoori, A. Hajari, A. A. Rownaghi, F. Rezaei, *ACS Appl. Mater. Interfaces* **2017**, *9*, 7489.
[24] N. Gargiulo, A. Verlotta, A. Peluso, P. Aprea, D. Caputo, *Microporous Mesoporous Mater.* **2015**, *215*, 1.
[25] Y. G. Ko, H. J. Lee, J. Y. Kim, U. S. Choi, *ACS Appl. Mater. Interfaces* **2014**, *6*, 12988.
[26] S. Couck, J. Lefevere, S. Mullens, L. Protasova, V. Meynen, G. Desmet, G. V. Baron, J. F. M. Denayer, *Chem. Eng. J.* **2017**, *308*, 719.
[27] S. Couck, J. Cousin-Saint-Remi, S. Van der Perre, G. V. Baron, C. Minas, P. Ruch, J. F. M. Denayer, *Microporous Mesoporous Mater.* **2018**, *255*, 185.
[28] F. A. Hasan, P. Xiao, R. K. Singh, P. A. Webley, *Chem. Eng. J.* **2013**, *223*, 48.
